# Evaluation of Staphylococcal Bacteriophage Sb-1 as an Adjunctive Agent to Antibiotics Against Rifampin-Resistant *Staphylococcus aureus* Biofilms

**DOI:** 10.3389/fmicb.2020.602057

**Published:** 2020-11-11

**Authors:** Lei Wang, Tamta Tkhilaishvili, Andrej Trampuz, Mercedes Gonzalez Moreno

**Affiliations:** ^1^Center for Musculoskeletal Surgery, Humboldt-Universität zu Berlin and Berlin Institute of Health, Corporate Member of Freie Universität Berlin, Charité – Universitätsmedizin Berlin, Berlin, Germany; ^2^BIH Center for Regenerative Therapies, Charité – Universitätsmedizin Berlin, Berlin, Germany

**Keywords:** rifampin-resistant *Staphylococcus aureus*, bacterial biofilm, antibiotic–bacteriophage combination, phage therapy, synergism, isothermal microcalorimetry

## Abstract

Rifampin plays a crucial role in the treatment of staphylococcal implant-associated infection, as it is the only antibiotic capable of eradicating *Staphylococcus aureus* biofilms. However, the emergence of rifampin resistance strongly limits its use. Combinatorial therapy of antibiotics and bacteriophages may represent a strategy to overcome the resistance. Here, we evaluated the activity of staphylococcal bacteriophage Sb-1 in combination with different antibiotics against the biofilms of 10 rifampin-resistant *S. aureus* clinical strains, including MRSA and MSSA. *S. aureus* biofilms formed on porous glass beads were exposed to antibiotics alone or combined with Sb-1 simultaneously or staggered (first Sb-1 for 24 h followed by antibiotic). Recovered bacteria were detected by measuring growth-related heat production at 37°C (isothermal microcalorimetry) and the biofilm eradication was assessed by sonication of beads and plating of the resulting sonication fluid. Minimum biofilm eradication concentration (MBEC) was defined as the lowest concentration of antibiotic required to kill all adherent bacteria, resulting in absence of growth after plating the sonication fluid. Tested antibiotics presented high MBEC values when administered alone (64 to > 1,024 μg/ml). The simultaneous or staggered combination of Sb-1 with daptomycin showed the highest activity against all MRSA biofilms, whereas the exposure to Sb-1 with vancomycin showed no improved anti-biofilm activity. Staggered administration of Sb-1 and flucloxacillin, cefazolin, or fosfomycin improved the antibiofilm activity in four out of six MSSA, whereas simultaneous exposure exhibited similar or lesser synergy. In conclusion, the combinatorial effect of Sb-1 and antibiotics enabled to eradicate rifampin-resistant *S. aureus* biofilms *in vitro*.

## Introduction

*Staphylococcus aureus* is one of the most common organisms causing implant-associated infections, such as periprosthetic joint infections (PJI), fracture-related infections (FRI), or spinal implant-associated infections (Tong et al., 2015). The pathogenesis involves the colonization of the device by microorganisms leading to the formation of biofilm on the surface of the implant, which makes the treatment of these infections challenging. Optimal treatment implies debridement and retention of the implant (in acute infections) or debridement with removal of devitalized material and exchange of implant that contain mature biofilm (in chronic infections) (Izakovicova et al., 2019). In both clinical situations, eradication of the biofilm with prolonged administration of biofilm-active antibiotics is required (Sendi et al., 2008).

The treatment of implant-associated infections due to *S. aureus* consists of initial intravenous antibiotic therapy, including nafcillin, oxacillin, flucloxacillin, cefazolin, or fosfomycin against methicillin-susceptible *S. aureus* (MSSA) and vancomycin, daptomycin, or fosfomycin against methicillin-resistant *S. aureus* (MRSA). In addition, rifampin is added to treat staphylococcal infections in patients who undergo debridement with retention or implant re-implantation in one- or two-stage exchange (Berbari et al., 2020). Rifampin should be co-administered with another active antibacterial agent since, otherwise, rifampin resistance emerges rapidly (Hoiby et al., 2015). With increased rifampin use, rifampin-resistant staphylococcal strains are increasing worldwide, representing an important concern. For example, in China, the rifampin resistance in MRSA isolates increased from 15.5 to 50.2% within 4 years (2004–2008) (Wang C. et al., 2019). Against rifampin-resistant mutants, rifampin has no biofilm activity *in vitro* or *in vivo* (Croes et al., 2010). Thus, alternative antimicrobial agents were investigated (e.g., daptomycin, fosfomycin, and dalbavancin), but none has shown biofilm activity *in vivo*. Another alternative is a combination of antibiotics with lytic bacteriophages. Lytic bacteriophages might exhibit rapid bactericidal activity, biofilm degradative properties, and the ability to enhance antibiotic activity (Tkhilaishvili et al., 2018) and are therefore considered as alternative strategies combating bacterial infections (Reardon, 2014).

Phage Sb-1 is one of the best characterized and fully sequenced lytic *staphylococcal* phage developed as an anti-infective therapy for human application by the Eliava Institute in Georgia (Kutateladze and Adamia, 2008). Its genome does not contain any bacterial virulence-associated genes, making it suitable for antimicrobial therapy (Kvachadze et al., 2011). Moreover, Sb-1 has been successfully used during the former Soviet Union to treat *S. aureus* infections in different patients (Sulakvelidze et al., 2001). However, there are limited numbers of *in vitro* and *in vivo* studies published regarding the activity of phage–antibiotic combination against *S. aureus* strains. Our previous study showed a good synergistic activity of phage Sb-1 and antibiotics against MRSA ATCC 43300 (Tkhilaishvili et al., 2018). In this study, we evaluated the efficacy of different classes of antibiotics (vancomycin, daptomycin, fosfomycin, gentamicin, flucloxacillin, cefazolin, and rifampin) alone or in combination with Sb-1, by either simultaneous or staggered application, against 10 rifampin-resistant *S. aureus* (RRSA) clinical strains (four MRSA and six MSSA) and the MRSA ATCC 43300 and MSSA ATCC 29213 laboratory strains.

## Materials and Methods

### Bacteria and Bacteriophage

Ten RRSA clinical isolates collected between 2015 and 2019 were included in this study. The clinical isolates were used from the biobank collection, which is part of the prospective institutional PJI cohort. The study was approved by the institutional ethical committee (EA1/040/14) and was conducted in accordance with the most recent iteration of the Declaration of Helsinki. According to the ethical approval, participants’ informed consent was waived, and all data were pseudonymized. Moreover, MRSA ATCC 43300 and MSSA ATCC 29213 laboratory standard strains were used in this study. Bacteria were stored at −80°C using a cryovial bead preservation system (Microbank; Pro-Lab Diagnostics, Canada). The staphylococcal phage Sb-1 was supplied by the Eliava Institute (Tbilisi, Georgia) and stored at 4°C.

### Antimicrobial Agents and Susceptibility Testing

Vancomycin (0.5 g, Hexal, Holzkirchen, Germany), daptomycin (0.5 mg, Novartis Pharma Schweiz, Basel, Switzerland), fosfomycin (5 g, InfectoPharm, Heppenheim, Germany), gentamicin injectable solution (40 mg/ml, Ratiopharm, Ulm, Germany), flucloxacillin (2 g, Stragen Pharma, Bad Homburg, France), cefazolin (2 g, MIP Pharma, Blieskastel-Niederwuerzbach, Germany), and rifampin (6 g, Sandoz Pharmaceuticals, Steinhausen, Switzerland) were provided from the respective manufacturers.

MIC for each antibiotic was determined by the broth macrodilution assay (BMD) in brain heart infusion broth (BHI; BD, Le Pont de Claix, France). An inoculum of approximately 5 × 10^5^ CFU/ml was used. Two fold serial dilutions of each antibiotic were prepared in sterile polystyrene round-bottom tubes to a final volume of 1 ml in inoculated medium and incubated for 24 h at 37°C. The MIC was defined as the lowest concentration of antibiotic that completely inhibited visible growth. BHI broth medium was supplemented with calcium chloride (40 μg/ml) and glucose 6-phosphate (25 μg/ml) when testing daptomycin and fosfomycin, respectively. All experiments were performed in triplicates.

The bacterial susceptibility to Sb-1 was evaluated in terms of efficacy of plating (EOP) as previously described (Wang et al., 2016). The EOP value was calculated as the ratio between the plaque-forming units (PFU) on the tested clinical strains with respect to the MRSA ATCC 43300 strain, defined as the host bacterium (EOP = phage titer on test bacterium/phage titer on host bacterium). EOP values of 0.5–1 were ranked as “high” efficiency; 0.2–0.5 as “medium” efficiency; 0.001–0.2 as “low” efficiency; 0.0 was considered as not effective against the target strain (Viazis et al., 2011).

### Evaluation of Antibiofilm Activity by Isothermal Microcalorimetry and Sonication/Colony Counting

The antibiofilm activity of single antibiotics and phage–antibiotic combination was determined by isothermal microcalorimetry (IMC), as previously reported (Tkhilaishvili et al., 2018). Briefly, biofilm formation was conducted by incubating porous glass beads (ROBU, Hattert, Germany) in inoculated BHI media at 37°C for 24 h. Beads where then washed (3x) with sterile 0.9% NaCl to remove planktonic cells and exposed to fresh BHI containing antibiotic. After 24 h of incubation at 37°C, beads were rinsed (3x) with 0.9% saline and inserted in microcalorimetry ampoules containing 3 ml of fresh BHI and introduced into the calorimeter. The minimum biofilm bactericidal concentration (MBBC) was defined as the lowest concentration of antibiotic that led to the absence of heat production after 48 h of incubation at 37°C. The effect of combined treatment (antibiotic + Sb-1) was evaluated by either simultaneous or staggered application, of 10^6^ PFU/ml Sb-1 phage and sub-MBBC concentrations of antibiotics. By simultaneous application, biofilms were exposed to antibiotics and Sb-1 during 24 h at 37°C. By staggered application, biofilms were exposed first to Sb-1 for 24 h and then to antibiotic for a further 24 h at 37°C. Evaluation of a staggered application of antibiotic followed by phage was discarded based on the unfavorable results observed in previous studies (Kumaran et al., 2018; Tkhilaishvili et al., 2018).

For samples where no heat production was detected, the complete biofilm eradication was determined by CFU counting of the sonicated beads after the microcalorimetric assay, and the minimum biofilm-eradicating concentration (MBEC) was defined as the lowest concentration of antibiotic required to kill all adherent bacteria, resulting in absence of any growth after plating of the sonication fluid (detection limit: <20 CFU/ml) (Gonzalez Moreno et al., 2019). All experiments were performed in triplicates.

The effect of phage–antibiotic combinations against biofilms was assessed as in a previous study (Ryan et al., 2012), determining the MBEC_phage_/MBEC_alone_ ratio, where MBEC_phage_ corresponds to the obtained MBEC value of an antibiotic tested in combination with the phage, and the MBEC_alone_ represents the obtained MBEC value of the same antibiotic when tested alone. Synergy was defined as a ratio ≤ 0.25, which correlated with a reduction of more than 2xMBEC_alone_. We combined and tested only concentrations of antibiotic that could reveal a synergistic effect with Sb-1 based on the MBEC values of the single antibiotic to be combined (antibiotics presenting an MBEC_alone_ > 1,024 μg/ml were tested in combination with Sb-1 at increasing concentrations up to 256 μg/ml).

## Results

### Bacterial Susceptibility to Conventional Antibiotics and Sb-1

The antimicrobial activity of antibiotics against planktonic and biofilm *S. aureus* was determined by BMD and by plating of sonication fluid, respectively. The obtained MIC and MBEC values are summarized in Table 1. Additionally, the MBBC values assessed by IMC and the effect of Sb-1 against the biofilm of both ATCC strains are shown in Supplementary Figures S1, S2.

**TABLE 1:** Antimicrobial susceptibility of planktonic (MIC) and biofilm (MBEC) *Staphylococcus aureus* strains determined by conventional broth macrodilution assay and sonication/colony-counting.

**Antibiotic**	**VAN**		**DAP**		**FOF**		**GEN**		**RIF**	
**MRSA strains**	**MIC**	**MBEC**	**MIC**	**MBEC**	**MIC**	**MBEC**	**MIC**	**MBEC**	**MIC**	**MBEC**
MRSA ATCC 43300	1	>1,024	0.5	128	8	>1,024	64(R)	>1,024	0.008	256
MRSA1	1	>1,024	0.5	64	4	>1,024	0.5	256	32(R)	>1,024
MRSA2	2	>1,024	1	128	4	>1,024	0.5	512	1(R)	>1,024
MRSA3	1	>1,024	0.5	64	128(R)	>1,024	0.5	512	32(R)	>1,024
MRSA4	1	>1,024	1	128	4	>1,024	0.5	>1,024	4(R)	>1,024

**Antibiotic**	**FLU**		**CFZ**		**FOF**		**GEN**		**RIF**	
**MSSA strains**	**MIC**	**MBEC**	**MIC**	**MBEC**	**MIC**	**MBEC**	**MIC**	**MBEC**	**MIC**	**MBEC**

MSSA ATCC 29213	0.5	>1,024	0.5	>1,024	4	>1,024	1	512	0.016	256
MSSA1	0.25	1,024	0.25	>1,024	16	>1,024	0.5	512	1(R)	>1,024
MSSA2	0.5	1,024	0.5	>1,024	4	>1,024	0.5	512	32(R)	>1,024
MSSA3	0.5	1,024	0.5	>1,024	8	>1,024	0.5	>1,024	32(R)	>1,024
MSSA4	0.25	>1,024	0.25	>1,024	8	>1,024	0.5	>1,024	1(R)	>1,024
MSSA5	0.5	512	0.5	>1,024	16	>1,024	8(R)	>1,024	2(R)	>1,024
MSSA6	0.5	>1,024	0.25	>1,024	4	>1,024	0.5	1,024	1(R)	>1,024

*MIC and MBEC concentration values are expressed in μg/ml. VAN, vancomycin; DAP, daptomycin; FOF, fosfomycin; GEN, gentamicin; RIF, rifampin; FLU, flucloxacillin; CFZ, cefazolin; R, resistance against the antibiotic according to EUCAST.*

Both ATCC strains were susceptible to all antibiotics according to the EUCAST breakpoints (EUCAST, 2020), except for MRSA ATCC 43300 that was resistant to gentamicin. The 10 RRSA strains were susceptible to all antibiotics, besides for MRSA4, resistant to fosfomycin, and MSSA5, resistant to gentamicin. All tested strains were susceptible to higher concentrations of antibiotics (ranging from 64 to > 1,024 μg/ml) when grown as biofilms if compared to the MIC values obtained for planktonic bacteria.

The antibiofilm activity of different antibiotics against the ATCC strains was evaluated by monitoring for 48 h the heat produced by biofilm bacteria still viable on the beads (after the exposure to the antibiotics) re-inoculated in fresh medium (Supplementary Figure S1). On the one hand, MRSA ATCC 43300 was susceptible to daptomycin and rifampin at concentrations of 128 and 256 μg/ml, respectively, whereas MSSA ATCC 29213 showed susceptibility to gentamicin and rifampin at concentrations of 512 and 256 μg/ml, correspondingly. The rest of the antibiotics, tested up to 1,024 μg/ml, showed no inhibition of heat flow production on the corresponding strain, indicating no antibiofilm activity despite the presence of high concentrations of antibiotic.

The exposure of the biofilm from both ATCC strains during 24 h to Sb-1 revealed a distinct effect on each strain. A remarkable reduction but not complete inhibition of the heat-flow production compared to the heat-flow produced by the growth control could be observed with the treated MRSA strain, whereas almost no difference between control and treated sample was observed for MSSA (Supplementary Figure S2). All *S. aureus* strains were susceptible to Sb-1 infection, showing EOP ratios ranging from 0.3 to 0.9 (Supplementary Table S1), indicative of a high lytic activity (EOP 0.5–1) of Sb-1 against most strains.

### Evaluation of Phage–Antibiotic Combinations Against ATCC Strains

**FIGURE 1:**
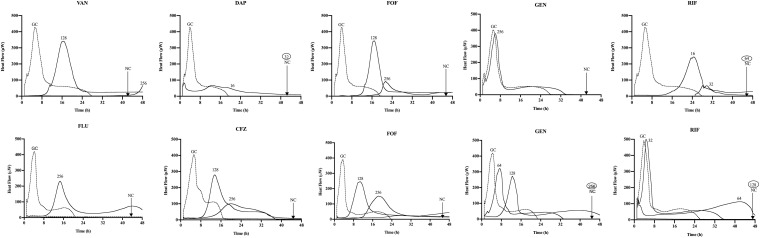
Microcalorimetry analysis of methicillin-resistant *Staphylococcus aureus* (MRSA) ATCC 43300 (upper row) and methicillin-susceptible *S. aureus* (MSSA) ATCC 29213 (bottom row) biofilms treated simultaneously with Sb-1 phage and sub-minimum biofilm bactericidal concentration (MBBC) concentrations of antibiotics. Each curve shows the heat produced by viable bacteria present in the biofilm after 24 h of phage–antibiotic treatment. Numbers represent concentrations (in μg/ml) of vancomycin (VAN), daptomycin (DAP), fosfomycin (FOF), gentamicin (GEN), rifampin (RIF), flucloxacillin (FLU), and cefazolin (CFZ). Circled values represent the MBBC, defined as the lowest antimicrobial concentration leading to absence of bacterial regrowth after 48 h. GC, growth control; NC, negative control. Data of a representative experiment are reported.

**FIGURE 2:**
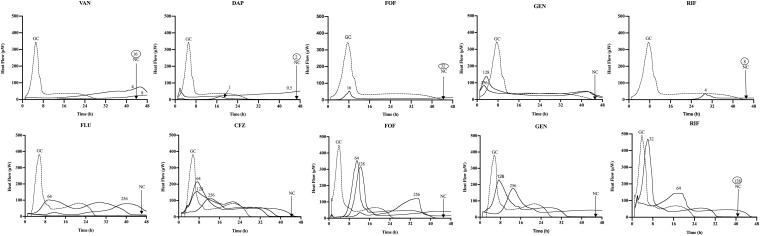
Microcalorimetry analysis of MRSA ATCC 43300 (upper row) and MSSA ATCC 29213 (bottom row) biofilms after staggered application of Sb-1 phage for 24 h followed by 24 h exposure to sub-MBBC concentrations of antibiotics. Each curve shows the heat produced by viable bacteria present in the biofilm after phage–antibiotic treatment. Numbers represent concentrations (in μg/ml) of vancomycin (VAN), daptomycin (DAP), fosfomycin (FOF), gentamicin (GEN), rifampin (RIF), flucloxacillin (FLU), and cefazolin (CFZ). Circled values represent the MBBC, defined as the lowest antimicrobial concentration leading to absence of bacterial regrowth after 48 h. GC, growth control; NC, negative control. Data of a representative experiment are reported.

The synergistic effect of simultaneous (Figure 1) and staggered (Figure 2) phage–antibiotic combinations against biofilm of both ATCC strains was investigated by IMC. Additionally, the presence of viable bacteria attached to the beads after calorimetry of those samples showing no heat production was evaluated by colony counting after bead sonication and plating of the sonication fluids. The obtained MBEC values as well as the calculated MBEC_phage_/MBEC_alone_ ratios are summarized in Table 2.

**TABLE 2:** Antibiofilm effects of simultaneous or staggered phage–antibiotic combinations.

**Antibiotic**	**Simultaneous exposure**		**Staggered exposure**	
	**MBEC (μg/ml)**	**Ratio (interpretation)**	**MBEC (μg/ml)**	**Ratio (interpretation)**
**MRSA ATCC 43300**				
VAN	>256	> 0.25(*NS*)^a^	16	0.015(S)
DAP	32	0.25(S)	2	0.015(S)
FOF	>256	> 0.25(*NS*)^a^	32	0.031(S)
GEN	>256	> 0.25(*NS*)^a^	>256	> 0.25(*NS*)^a^
RIF	64	0.25(S)	8	0.031(S)
**MSSA ATCC 29213**				
FLU	>256	> 0.25(*NS*)^a^	>256	> 0.25(*NS*)^a^
CFZ	>256	> 0.25(*NS*)^a^	>256	> 0.25(*NS*)^a^
FOF	>256	> 0.25(*NS*)^a^	>256	> 0.25(*NS*)^a^
GEN	256	0.5(*NS*)	>256	> 0.25(*NS*)^a^
RIF	128	0.5(*NS*)	128	0.5(*NS*)

*VAN, vancomycin; DAP, daptomycin; FOF, fosfomycin; GEN, gentamicin; RIF, rifampin; FLU, flucloxacillin; CFZ, cefazolin; S, synergism; NS, no synergism. ^*a*^MBEC value above 1/4xMBEC_*alone*_ (considering MBEC_*alone*_ equal to 1,024 μg/ml), thus MBEC_*phage*_/MBEC_*alone*_ ratio is interpreted as >0.25 (NS).*

Among all simultaneously tested phage–antibiotic combinations against MRSA ATCC 43300, only the exposure of biofilm to Sb-1 and sub-MBBC concentrations of daptomycin or rifampin showed a synergistic effect. In contrast, the strongest synergistic effect was observed by staggered exposure of MRSA ATCC 43300 to Sb-1 and sub-MBBC concentrations of vancomycin or daptomycin, showing the lowest MBEC_phage_/MBEC_alone_ ratios, followed by fosfomycin and rifampin (Table 2), whereas no synergistic effect was observed with gentamicin, possibly due to the resistance profile of this strain toward gentamicin.

No synergistic effect was observed by the phage–antibiotic combinations against MSSA ATCC 29213, either simultaneous or staggered. Overall, when MRSA ATCC 43300 biofilm was first exposed to Sb-1 during 24 h prior to the exposure to sub-MBBC concentrations of antibiotics, a higher delay and/or reduction of heat-flow production was obtained compared to the heat-flow produced when biofilm was exposed to simultaneous phage/antibiotic combinations at the same antibiotic concentrations, whereas this effect was not as noteworthy in the case of MSSA ATCC 29213.

### Evaluation of Phage–Antibiotic Combinations Against Clinical Rifampin-Resistant *Staphylococcus aureus* Strains

The ability of phage–antibiotic combinations to eradicate the biofilm of 10 clinical rifampin-resistant MRSA and MSSA strains was directly evaluated by sonication/colony-counting as described previously, and results are shown in Table 3.

**TABLE 3:** Antibiofilm effects of simultaneous (MBEC_*SIM*_) or staggered (MBEC_*STA*_) phage–antibiotic combinations against clinical strains.

**Antibiotic**	**VAN**		**DAP**		**FOF**		**GEN**	
**MRSA strains**	**MBEC_SIM_**	**MBEC_STA_**	**MBEC_SIM_**	**MBEC_STA_**	**MBEC_SIM_**	**MBEC_STA_**	**MBEC_SIM_**	**MBEC_STA_**
MRSA1	>256 (NS)^a^	>256 (NS)^a^	8 (0.125, S)	8 (0.125, S)	>256 (NS)^a^	64 (0.06, S)^b^	64 (0.25, S)	64 (0.25, S)
MRSA2	>256 (NS)^a^	>256 (NS)^a^	16 (0.125, S)	16 (0.125, S)	>256 (NS)^a^	256 (0.25, S)^b^	256 (0.5, NS)	64 (0.125, S)
MRSA3	>256 (NS)^a^	>256 (NS)^a^	16 (0.25, S)	16 (0.25, S)	>256 (NS)^a^	>256 (NS)^a^	128 (0.25, S)	64 (0.125, S)
MRSA4	>256 (NS)^a^	>256 (NS)^a^	16 (0.125, S)	16 (0.125, S)	>256 (NS)^a^	>256 (NS)^a^	>256 (NS)^a^	>256 (NS)^a^

**Antibiotic**	**FLU**		**CFZ**		**FOF**		**GEN**	
**MSSA strains**	**MBEC_SIM_**	**MBEC_STA_**	**MBEC_SIM_**	**MBEC_STA_**	**MBEC_SIM_**	**MBEC_STA_**	**MBEC_SIM_**	**MBEC_STA_**

MSSA1	128 (0.125, S)	64 (0.006, S)	>256 (NS)^a^	256 (0.25, S)^b^	>256 (NS)^a^	>256 (NS)^a^	128 (0.25, S)	128 (0.25, S)
MSSA2	256 (0.25, S)	32 (0.003, S)	>256 (NS)^a^	128 (0.125, S)^b^	>256 (NS)^a^	64 (0.006, S)^b^	128 (0.25, S)	128 (0.25, S)
MSSA3	256 (0.25, S)	32 (0.003, S)	>256 (NS)^a^	256 (0.25, S)^b^	>256 (NS)^a^	256 (0.25, S)^b^	>256 (NS)^a^	>256 (NS)^a^
MSSA4	>256 (NS)^a^	>256 (NS)^a^	>256 (NS)^a^	>256 (NS)^a^	>256 (NS)^a^	256 (0.25, S)^b^	>256 (NS)^a^	>256 (NS)^a^
MSSA5	256 (0.5, NS)	256 (0.5, NS)	>256 (NS)^a^	>256 (NS)^a^	>256 (NS)^a^	>256 (NS)^a^	>256 (NS)^a^	>256 (NS)^a^
MSSA6	>256 (NS)^a^	32 (0.003, S)^b^	>256 (NS)^a^	16 (0.015, S)^b^	>256 (NS)^a^	32 (0.003, S)^b^	256 (0.25, S)	256 (0.25, S)

*MBEC concentration values are expressed in μg/ml. In brackets is shown the ratio value followed by the ratio interpretation. VAN, vancomycin; FLU, flucloxacillin; DAP, daptomycin; CFZ, cefazolin; FOF, fosfomycin; GEN, gentamicin; S, synergism; NS, no synergism. ^*a*^MBEC value above 1/4xMBEC_*alone*_ (considering MBEC_*alone*_ equal to 1,024 μg/ml), thus MBEC_*phage*_/MBEC_*alone*_ ratio is interpreted as >0.25 (NS). ^*b*^MBEC_*alone*_ considered equal to 1,024 μg/ml for MBEC_*phage*_/MBEC_*alone*_ ratio calculations.*

Among four MRSA isolates, a synergistic effect was observed for all strains after exposure to Sb-1/daptomycin combination (either simultaneously or staggered), in three strains (75%) when exposure to Sb-1/gentamicin staggered combination and in two (50%) strains exposed to staggered Sb1/fosfomycin or to simultaneous Sb-1/gentamicin combination. No synergistic effect was observed when the biofilm of tested clinical strains was exposed to either simultaneous or staggered Sb-1/vancomycin combination, in contrast to the finding with MRSA ATCC 43300 strain.

Among six MSSA isolates, synergistic effect was observed in four strains (67%) after staggered exposure to Sb-1/flucloxacillin or to Sb-1/cefazolin, and in three strains (50%) with simultaneous Sb-1/flucloxacillin combination. Only staggered but not simultaneous Sb-1/fosfomycin combination revealed a synergistic effect against four strains (67%), whereas simultaneous or staggered Sb-1/gentamicin combination showed synergism against three strains (50%). None of the tested simultaneous or staggered phage/antibiotic combinations presented an improvement in the antimicrobial activity compared to the action of each antimicrobial agent alone against MSSA5 biofilm.

Moreover, no synergism was found with Sb-1 and rifampin combination against MRSA or MSSA (Supplementary Table S2).

## Discussion

Biofilm formation on the device surface is the key occurrence in the pathogenesis of implant-associated infections, requiring the use of biofilm-active antibiotics (Davidson et al., 2019). Rifampin emerged about three decades ago as an antibiofilm antibiotic against *S. aureus* orthopedic implant-associated infections (Zimmerli and Sendi, 2019), presenting good penetration and bioavailability in osteo-articular tissue (Sendi and Zimmerli, 2017). In this study, we investigated alternatives to rifampin for the treatment of implant-associated infections caused by RRSA.

Phage efficacy has been described to be influenced by host specificity, among several other factors (Ly-Chatain, 2014). In our study, Sb-1 showed high killing effect against most tested strains, but still a complete biofilm eradication with the phage alone was not achieved, possibly due to the establishment of an equilibrium between virus and host, as reported earlier (Głowacka-Rutkowska et al., 2019), what might be prevented with the addition of antibiotics.

The phage–antibiotic combinations tested in our study were selected based on the methicillin-resistant profile of the *S. aureus* isolates, as usually done in the clinical setting (Berbari et al., 2020). Hence, in addition to testing fosfomycin and gentamicin against all strains, daptomycin and vancomycin were selected for testing on MRSA strains, while flucloxacillin and cefazolin were selected for testing on MSSA strains. For the evaluation of phage–antibiotic combinations, a fixed value of 1,024 μg/ml was considered for the calculation of the MBEC_phage_/MBEC_alone_ ratios for samples with MBEC_alone_ > 1,024 μg/ml. It should be noted that, by this approach, some combinations that were interpreted as not synergistic could turn out to have a synergistic effect when testing higher MBEC values. However, the observed positive synergistic effects of phage–antibiotic combinations with our experimental setup are certain and usually presenting considerably lower MBEC values compared to the MBEC values of single antibiotics.

The determination of the EOP ratios is a frequent test to identify phages suitable for phage therapy (Khan Mirzaei and Nilsson, 2015). In our study, however, we did not observe a correlation between the EOP rank and the antibiofilm activity of the phage against a specific strain. For instance, Sb-1 showed low efficacy against MSSA ATCC 29213 biofilm with no synergistic effect in combination with antibiotics despite a high EOP rank, but Sb-1 in combination with daptomycin resulted in a synergistic effect against MRSA3 and MRSA4 although showing lower EOP ratios on these strains. Thus, in the context of using phages to control bacterial biofilms, the determination of the EOP ratios should not be misinterpreted toward a correlation to efficiency against biofilms. The nature of the biofilm matrix can differ among strains, ultimately affecting the bioavailability and/or function of an antimicrobial, as suggested by Bauer and coworkers (Bauer et al., 2013), who evaluated antibiotic activity on young and mature MSSA and MRSA biofilms and observed that, besides biofilm maturity, the bacterial strain clearly influenced antibiotic activity.

The order of administration when combining antibiotics and phages has been shown to play a key role for a synergistic antimicrobial effect (Dickey and Perrot, 2018; Kumaran et al., 2018). We observed a synergistic effect when Sb-1 was combined with fosfomycin or cefazolin by staggered application but not when these antibiotics and Sb-1 were applied simultaneously. Moreover, the pre-exposure to Sb-1 followed by flucoxacillin eradicated the biofilm at lower antibiotic concentrations compared to simultaneous application. These findings indeed seem to indicate that exposure of biofilms first to phage followed by antibiotics is the most effective way to eradicate them. Previous studies have shown the benefit of the staggered application when combining antibiotics and phages (Tkhilaishvili et al., 2018), while a simultaneous exposure could result in hindering their antibiofilm efficacy, possibly due to antagonistic modes of action (e.g., antibiotics interfering with the bacterial DNA replication process) or due to the killing of host bacteria – which is essential for phage production – by the antibiotic (Chaudhry et al., 2017; Kumaran et al., 2018; Akturk et al., 2019).

On the other hand, other than with the MRSA ATCC strain, combining Sb-1 and daptomycin or vancomycin against rifampin-resistant MRSA strains exhibited the same outcome independently of the order of administration. In a previous study by Dickey and Perrot (2018), the authors also showed that a simultaneous treatment of *S. aureus* biofilm with daptomycin and phage was as effective as sequential treatment. Moreover, they showed an antagonistic effect when combining phage and vancomycin. Considering that the wall teichoic acid from the bacterial cell wall is the primary staphylococcal phage receptor (Azam and Tanji, 2019) and that vancomycin has a unique mechanism of action inhibiting cell wall synthesis (Watanakunakorn, 1984), it is conceivable that phage infection was negatively affected by the vancomycin impact on the bacterial cell wall. Daptomycin action disrupting the bacterial cell membrane structure seems to have a lower interference with phage action. As shown also by Dickey and Perrot, the simultaneous application of phage and daptomycin at 10xMIC allowed phage growth, whereas most antibiotics tested in their study at 10xMIC either prevented phage growth (ciprofloxacin, vancomycin, and tetracyclin) or led to massive decreases in phage density (gentamicin, erythromycin, and linezolid) (Dickey and Perrot, 2018).

Generally, vancomycin is recommended for the treatment of MRSA implant-associated infections (Paiva and Eggimann, 2017), yet a high rate of vancomycin treatment failure in vancomycin-susceptible MRSA infections has been reported (Dombrowski and Winston, 2008; Abdelhady et al., 2013). This observation correlates with our findings showing the inefficiency of treating biofilms of clinical strains with vancomycin alone or combined with Sb-1. The low efficacy of vancomycin against staphylococcal biofilms could be due to a reduced biofilm penetration, a reduced concentration of free vancomycin being sequestrated by *S. aureus* on peptidoglycan layers, or a stimulation of biofilm formation by low concentrations of vancomycin (Broussou et al., 2018). Conversely, daptomycin has shown a superior efficacy against bone and joint infections caused by MRSA (Chang et al., 2017; Telles et al., 2019). The consistent synergistic effect observed with Sb-1/daptomycin combination against all tested rifampin-resistant MRSA strains in our study, together with the good biofilm penetration properties (Ozturk et al., 2016) and *in vitro* activity against stationary-phase bacteria inside the biofilm (Smith et al., 2009), makes daptomycin a promising candidate for combinatorial therapy.

Another promising therapeutic approach based on our results was found by the combination of Sb-1 with flucloxacillin for the eradication of rifampin-resistant MSSA strains, where there was a remarkable reduction in the MBEC values, and MBEC_phage_/MBEC_alone_ ratios as low as 0.003, could be observed after staggered phage-antibiotic administration against 67% of the strains. Analysis on the production of type A beta-lactamase by MSSA strains, responsible for cefazolin hydrolysis (Nannini et al., 2009), could bring insights on the lower antibiofilm efficacy of cefazolin compared to flucloxacillin.

Low MBEC_phage_/MBEC_alone_ ratios (ranging from 0.003 to 0.06) were also observed with staggered administration of Sb-1 with fosfomycin. Fosfomycin has been shown to act synergistically with other antibiotics against biofilms of different bacterial species, including MRSA, in part probably because of its broad-spectrum bactericidal activity (Chai et al., 2016; Wang L. et al., 2019). In addition, favorable characteristics associated to fosfomycin include the ability to break up biofilms and enhance the permeability of other antimicrobials and a presumed immunomodulatory effect (Mihailescu et al., 2014).

Previous studies revealed synergistic effects by combining phage with an antibiotic for which the bacteria strain was resistant (Liu et al., 2020). In our study, however, the use of Sb-1 in combination with rifampin against biofilms of RRSA strains, as well as Sb-1/fosfomycin and Sb-1/gentamicin combinations against MRSA3 and MRSA ATCC 43300, respectively, did not reveal an improved anti-biofilm effect.

When trying to draw conclusions or make clinical extrapolations, it is important to consider the small number of tested strains in our study. We aimed to provide the first original data on the combinatorial effect of Sb-1 and different antibiotics to eradicate RRSA biofilms *in vitro.* Our work highlights that findings obtained testing ATCC strains may differ from the outcome with clinical isolates, but also among the different strains, implying that selecting an appropriate phage–antibiotic combinatorial therapy will be highly dependent on the strain causing the infection as well as on the specific antibiofilm efficacy of the phage, more than its lytic spectrum. Moreover, there is substantial evidence that many antibiotics can interfere with phage infection activity—especially at concentrations exceeding measured minimum inhibitory concentrations—and thus with phage primary pharmacodynamic properties. Hence, further preclinical and clinical studies are essential to support the development of phage/antibiotic combination therapy with particular isolates. Factors that point toward a more personalized approach for the successful treatment of antibiotic-resistant implant-associated infections.

## Data Availability Statement

The original contributions presented in the study are included in the article/Supplementary Material, further inquiries can be directed to the corresponding author.

## Author Contributions

LW and TT performed the experiments with the contribution of MG. MG, LW, and TT analyzed the data. MG and TT drafted the manuscript with the contribution of LW and AT. All authors conceived and designed the experiments and revised and approved the final version of this manuscript.

## Conflict of Interest

The authors declare that the research was conducted in the absence of any commercial or financial relationships that could be construed as a potential conflict of interest.
