# Supplementary material for: Evaluation of Staphylococcal Bacteriophage Sb-1 as an Adjunctive Agent to Antibiotics Against Rifampin-Resistant Staphylococcus aureus Biofilms
Source: Front Microbiol. 2020 Nov 11;11:602057. doi: 10.3389/fmicb.2020.602057 (PMC7686474; doi:10.3389/fmicb.2020.602057)
Supplement: Supplementary file 1 [file Data_Sheet_1.docx]

Supplementary Material

Evaluation of staphylococcal bacteriophage Sb-1 as an adjunctive agent to antibiotics against rifampin-resistant *Staphylococcus aureus* biofilms

Lei Wang, Tamta Tkhilaishvili, Andrej Trampuz and Mercedes Gonzalez Moreno

**Correspondence:**Mercedes Gonzalez Moreno
mercedes.gonzalez-moreno@charite.de

**
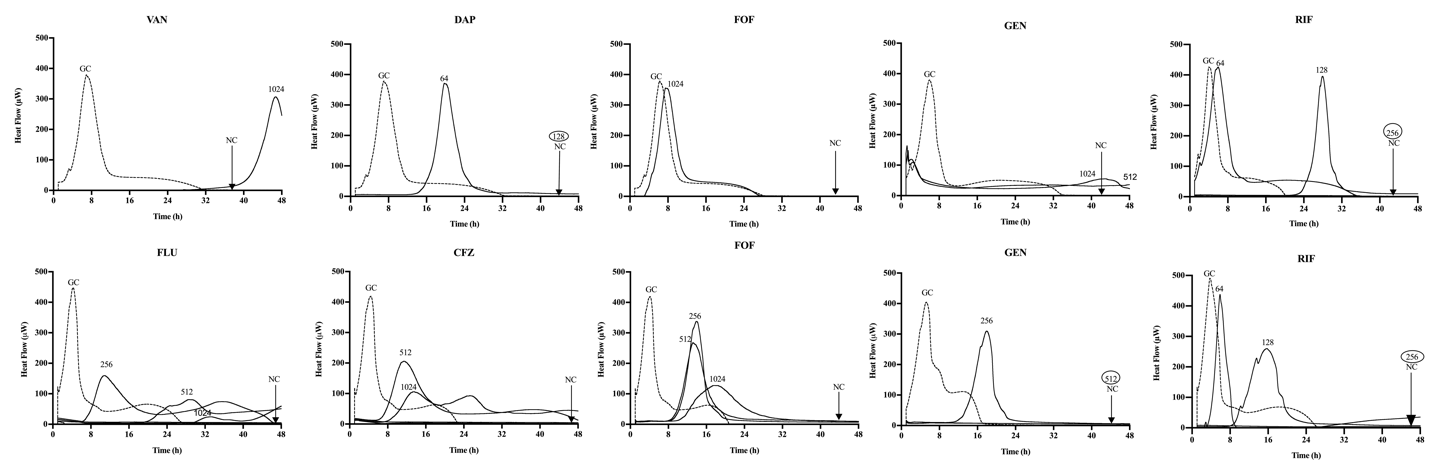
Figure S1.** Microcalorimetry analysis of MRSA ATCC 43300 (upper row) and MSSA ATCC 29213 (bottom row) biofilms treated with different antibiotic concentrations. Each curve shows the heat produced by viable bacteria present in the biofilm after 24h of antibiotic treatment or no treatment. Numbers represent concentrations (in µg/ml) of vancomycin (VAN), daptomycin (DAP), fosfomycin (FOF), gentamicin (GEN), rifampin (RIF), flucloxacillin (FLU) and cefazolin (CFZ). Circled values represent the MBBC, defined as the lowest antimicrobial concentration leading to absence of bacterial regrowth after 48 h. GC, growth control; NC, negative control. Data of a representative experiment are reported.





**Figure S2.** Microcalorimetry analysis of MRSA ATCC 43300 (right plot) and MSSA ATCC 29213 (left plot) biofilms treated with Sb-1 (10^6^ PFU/ml). Each curve shows the heat produced by viable bacteria present in the biofilm after 24h of exposure to Sb-1 (continuous line) or no exposure (dotted line). GC, growth control; NC, negative control. Data of a representative experiment are reported.

**Table S1.** Determination of the efficiency of plating (EOP) of Sb-1 phage dilutions against target bacteria. EOP values were determined using MRSA ATCC 43300 as reference strain. Values are expressed as average ± standard deviation.

| **Strain** | **EOP** | **Rank** |
| --- | --- | --- |
| MRSA1 | 0.58 ± 0.03 | high |
| MRSA2 | 0.81 ± 0.06 | high |
| MRSA3 | 0.49 ± 0.01 | medium/high |
| MRSA4 | 0.38 ± 0.01 | medium |
| MSSA1 | 0.75 ± 0.05 | high |
| MSSA2 | 0.93 ± 0.04 | high |
| MSSA3 | 0.66 ± 0.12 | high |
| MSSA4 | 0.68 ± 0.05 | high |
| MSSA5 | 0.57 ± 0.07 | high |
| MSSA6 | 0.94 ± 0.02 | high |
| MSSA ATCC 29213 | 0.73 ± 0.004 | high |

EOP values of 0.5–1 ranked as ‘high’ efficiency; 0.2–0.5 as ‘medium’ efficiency;

0.001–0.2 as ‘low’ efficiency; 0.0 was considered as not effective against the target strain.

**Table S2.** Anti-biofilm effects of simultaneous (MBEC_SIM_) or staggered (MBEC_STA_) Sb-1/rifampin combinations against clinical strains. MBEC concentration values are expressed in µg/ml. In brackets is shown the ratio interpretation.

|  | **RIF** | |
| --- | --- | --- |
| **MRSA strains** | **MBEC_SIM_** | **MBEC_STA_** |
| MRSA1 | ＞256^a^ (NS) | ＞256^a^ (NS) |
| MRSA2 | ＞256^a^ (NS) | ＞256^a^ (NS) |
| MRSA3 | ＞256^a^ (NS) | ＞256^a^ (NS) |
| MRSA4 | ＞256^a^ (NS) | ＞256^a^ (NS) |
| **MSSA strains** | **MBEC_SIM_** | **MBEC_STA_** |
| MSSA1 | ＞256^a^ (NS) | ＞256^a^ (NS) |
| MSSA2 | ＞256^a^ (NS) | ＞256^a^ (NS) |
| MSSA3 | ＞256^a^ (NS) | ＞256^a^ (NS) |
| MSSA4 | ＞256^a^ (NS) | ＞256^a^ (NS) |
| MSSA5 | ＞256^a^ (NS) | ＞256^a^ (NS) |
| MSSA6 | ＞256^a^ (NS) | ＞256^a^ (NS) |

RIF, rifampin; NS, no synergism

^a^MBEC value above 1/4xMBEC_alone_ (considering MBEC_alone_ equal to 1024 µg/ml),

thus MBEC_phage_/MBEC_alone_ ratio is interpreted as >0.25 (NS).
